# Application of a New Established System for Toxic Doses in Children With 4-Hydroxycoumarin Rodenticide Intoxication

**DOI:** 10.3389/fped.2018.00141

**Published:** 2018-05-15

**Authors:** Li Ye, Zheng Wang, Hui Zhang, Hui Guo, Yannan Guo, Lina Chen

**Affiliations:** ^1^Department of Pediatrics, West China Second University Hospital of Sichuan University, Chengdu, China; ^2^Key Laboratory of Obstetric and Gynecologic and Pediatric Diseases and birth Defects of Ministry Education, West China Second University Hospital of Sichuan University, Chengdu, China

**Keywords:** 4-hydroxycoumarins, rodenticide, poisoning, child, Vitamin K_1_, acquired coagulopathy

## Abstract

The toxic dose of rodenticides in children is extremely difficult to be determined because of the uncertain exposure history. We established and validated a method to identify the toxic dose in children of 4-hydroxycoumarin (TDCH). Items were selected by Delphi method and weighted by analytic hierarchy process. Toxic doses were classified into three categories: high dose (>24 points), medium (15-24) and low (<15). Sixty-five children with 4-hydroxycoumarin rodenticide intoxication were included in the study. There were 29(44.6%), 8(12.3%), 28(43.1%) cases in high, medium, and low dose respectively. Patients in high-dose were more likely to have intentionally attempted suicide (5/29, 17.2%) or had no definite history of ingestion (17/29, 58.6%), arrived at the hospital later than 24 h (26/29, 90%), been misdiagnosed initially (25/29, 86.2%), not treated by gastric lavage (27/29, 93.1%), and developed severe hemorrhage. While most patients in low-dose were younger than 6 years (26/28, 92.9%), all have experienced accidental exposure, arrived at the hospital, and received gastric lavage within 24 h, obtained a definite diagnosis, and be asymptomatic. Of 38 patients arrived at hospital within 48 h, patients a score_48h_ ≥ 15 had higher incidence of coagulopathy (6/8, 75.0%) than patients with a score_48h_ < 15 (3/30, 10.0%). Of all patients, 37 in the high and medium dose with a score ≥ 15 has higher incidence (35/37, 94.6%) of prolonged administration with vitamin K_1_ (≥1 month) than other 28 patients with a score < 15 (0/28, 0%). The TDCH system could not only be used in evaluating toxic doses and predicting coagulopathy in the early stage, but also helps to guide appropriate treatment. Patients with a score_48h_ ≥ 15 were in the high bleeding risk category. And patients with a scores ≥ 15 required treatment with vitamin K_1_ for more than a month.

## Introduction

Since the mid-1970s, the widespread use of 4-hydroxycoumarin instead of short-acting rodenticides (e.g., arsenic and strychnine) has increased the incidence of long-acting anticoagulant rodenticides (LAARs) poisoning (1). Currently, the majority of commercially available LAARs are second-generation 4-hydroxycoumarin such as brodifacoum and bromadiolone that have increased activity and a longer duration of action than those of the parent compound (2, 3). Thus, they are also referred to as “superwarfarins.” 4-Hydroxycoumarin inhibits the activity of hepatic vitamin K1 epoxide reductase, which is involved in the synthesis of the active form of vitamin K, and subsequently vitamin K-dependent blood clotting factors II, VII, IX, and X (4). The mean half-life of factors II, VII, IX, and X is 41.0, 6.2, 13.9, and 16.5 h, respectively (5). Therefore, after ingestion of 4-hydroxycoumarin, coagulopathy may be delayed by several hours to days until the preformed vitamin K and active clotting factors are exhausted.

Clinical manifestations of 4-hydroxycoumarin poisoning are characterized by prolonged multiple-site hemorrhage, and may range from no symptom to life-threatening bleeding, which is mainly determined by the ingested dose of the active ingredient. Significant coagulopathy usually results from the ingestion of large amounts of this active ingredient without timely diagnosis and treatment. And clinically important manifestation would not be observed in children who ingested less than 1 box of superwarfarin (6). However, in contrast to that in adults, the ingested dose of rodenticides in children is very difficult to be determined because of the uncertainties in the exposure history (7). Preschool children who comprise the largest at-risk population of rodenticide poisoning are usually unable to accurately describe the poisoning details including ingested doses. Suicidal adolescents, for various reasons, may intentionally conceal the facts. Meanwhile, some guardians do not witness the process of rodenticide ingestion. Furthermore, 4-hydroxycoumarin is usually unnoticed because it is flavorless (8). Some consumers use the concentrates to prepare bait in a manner not strictly in accordance with the instructions and, therefore, the amount of the active substance ingested is unknown.

These domestic and household challenges make it extremely difficult to estimate the ingested dose accurately. When the ingested dose is known, decontamination and treatment can increase the elimination of toxins resulting in the decrease of toxic *in vivo* dose, and vitamin K administration will decrease the toxic effects. As a result, toxic dose is not always consistent with ingested dose. To date, there are very little data available on the toxic dose estimation of 4-hydroxycoumarin in humans. Therefore, the following study was conducted to establish an evaluation system for toxic doses in pediatric patients with 4-hydroxycoumarin rodenticide intoxication. Using this evaluation system, we aimed to guide the accurate administration of poisoned patients.

## Material and methods

### Study design

A Delphi group consisted of 12 pediatric experts and 8 toxicologists were established to aid with item selection and gradation. A systematic review of the literature was performed using the following search items: “4-hydroxycoumarin,” “coumarins,” “anticoagulant,” “long-acting anticoagulant,” “anticoagulant rodenticide,” “superwarfarin,” “brodifacoum,” and “bromadiolone.” Databases queried included PubMed, Science Citation Index, and OVID. 10 items (age, amount ingested, reason, LAARs type, coagulation function, severity of bleeding, time from ingestion to first-time gastric lavage, time from ingestion to first-time plasmapheresis, time from ingestion to hospitalization, and frequency of plasmapheresis) which closely related to toxic dose and severity of disease were initially drafted through review of the literature. The Delphi group members ranked each item from 0(not important) to 4(extremely important) separately. The responses of all Delphi members were summarized, processed, and sent back to the experts. Consensus was reached after two rounds and 7 items (amount ingested, reason, coagulation function, severity of bleeding, time from ingestion to first-time gastric lavage, time from ingestion to first-time plasmapheresis and frequency of plasmapheresis) were selected finally. Then gradations were weighted by analytic hierarchy process. Finally, the scoring system for TDCH was established according to item weighting.

Trained physicians recorded the information, and prothrombin time (PT), activated partial prothrombin time (APTT), and international normalized ratio (INR) were measured for all patients on admission, and toxic dose grades of all patients were made by the TDCH scores. All patients were routinely followed up, and PT, APTT, and INR repeated at a month. For patients who needed vitamin K1 treatment more than a month, they were further followed until recovery. The ability of TDCH score_48h_ predicting coagulopathy and TDCH score predicting prolonged administered with vitamin K_1_ was assessed with the area under the receiver operating characteristic (AUROC) analysis. In 38 patients who arrived at the hospital within 48 h, TDCH score_48h_ were obtained according to manifestations and lab findings. And prolonged administration with vitamin K_1_ was defined as longer than a month.

### Patients

Sixty-five children at the West China Second University Hospital of Sichuan University, who were diagnosed with 4-hydroxycoumarin rodenticide intoxication between May 2008 and September 2016, were included in the study. The diagnosis of 4-hydroxycoumarin rodenticide intoxication was based on exposure history, clinical effects, physical examination, and laboratory results. Main hemorrhagic symptoms of the enrolled patients included epistaxis, petechiae, gastro- intestinal bleeding, hematuria, ecchymoma, and gingival bleeding. In 17 cases who lacked a definitive history of ingestion, the diagnoses were confirmed using high-performance liquid chromatography with tandem mass spectrometry (LC-MS/MS) detection of blood samples (9). The following were the study exclusion criteria: (1) combined ingestion of other pesticides or anticoagulants such as warfarin; (2) hereditary coagulation deficiency; and (3) history of serious underlying disease such as heart, lung, renal, or liver disease.

### Statistical analysis

The statistical package for the social sciences (SPSS) version 19.0 software was used for the statistical analyses. The measured data were expressed as the mean ± standard deviation (*SD*) and counted data were expressed as numbers and percentages in parentheses. The differences between groups were compared using the Student's *t*-test for continuous variables and the chi-square test for categorical variables. The Delphi method was used to select items. Analytic hierarchy process was used to determine the weight of each item. AUROC analysis was used to calculate cutoff values, sensitivity, and specificity. Differences with *p-*values < 0.05 were considered statistically significant.

### Ethics statements

This study was performed according to the Declaration of Helsinki and was approved by the Medical Ethics Review Committee of West China Second University Hospital of Sichuan University (reference number 2017032). All parents of participants signed an informed consent form prior to enrolment.

## Results

### Scoring system for toxic doses in children with 4-hydroxycoumarin (TDCH)

The specific calculation of the evaluation system is shown in Table 1.

**Table 1:** Scoring system for toxic dose in children with 4-hydroxycoumarin (TDCH).

**Contributing factors**		**Scores**
**AMOUNT INGESTED**^a^		
**Concentrate (mL)**	**Bait (g)**	
<0.2	<10	12
0.2–2	10–30	16
2–5	30–50	20
≥5	≥50	24
**REASON**^a^ **(OPTIONAL)**		
Accidental		16
Unknown^b^		20
Suicide		24
**LABORATORY EXAMINATIONS**		
INR	APPT	
<1.5	Normal	0
1.5–3	>100–150% increase from baseline	2
3–5	>150–250% increase from baseline	4
≥5	≥250% increase from baseline	6
**BLEEDING**^c^		
No		0
Mild		2
Moderate		4
Severe		6
**Weakening factors**		**Scores**
**TIME FROM INGESTION TO FIRST-TIME GASTRIC LAVAGE (HOURS)**		
≥24 or no		0
4–24		−2
1–4		−4
<1		−6
**TIME FROM INGESTION TO FIRST-TIME PLASMAPHERESIS (DAYS)**		
no		0
≥7		−2
1–7		−4
<1		−6
**FREQUENCY OF PLASMAPHERESIS**		
0		0
1		−1
2–3		−2
≥4		−3

*The total score of toxic dose was obtained by summing up the points of contributing factors and weakening factors. High dose group: >24 points; medium: 15–24 points; low: < 15 points*.

a *When the amount ingested was clear, the score was obtained according to amount ingested. When the amount ingested was uncertain, the score was obtained according to poisoning reason*.

b *Reasons for poisoning were unknown because exposure history was unavailable*.

c *No, no bleeding at all; mild, bruising, petechiae, occasional mild epistaxis with very little or no interference with daily living; moderate, more severe skin manifestations with some mucosal lesions and more troublesome epistaxis or menorrhagia; severe, bleeding episodes (epistaxis, melena, menorrhagia and/or intracranial hemorrhage) requiring hospital admission and/or blood transfusions (10). TDCH, toxic dose in children with 4-hydroxycoumarin; INR, international normalized ratio; APPT, activated partial thromboplastin time*.

### Baseline characteristics

As shown in Table 2, 65 children with 4-hydroxycoumarin intoxication aged between 9 months and 15 years were included in the study. The pediatric 4-hydroxycoumarin poisoning cases exhibited a bimodal age distribution. Poisoning Reasons of 17 patients were unknown because their exposure history was unavailable, of whom, 9 repeatedly denied any exposure to rodenticides, and other 8 denied the ingestion despite evidence of the use of rodenticides in their household. The diagnosis of them was confirmed by LC-MS/MS method. Epistaxis (23/65) and petechiae (21/65) were main hemorrhagic symptoms, followed by gastro- intestinal bleeding (16/65), hematuria (7/65), ecchymoma (7/65) and gingival bleeding (6/65). Mortality rate was zero.

**Table 2:** Baseline characteristics of 65 children with 4-hydroxycoumarin intoxication.

**Variable**	**N (%) or Mean (*SD*)**
**AGE (YEARS)**	
<6	44 (67.7)
6– <10	8 (12.3)
≥10	13 (20.0)
**SEX**	
Male	35 (53.8)
Female	30 (46.2)
**ENVIRONMENT**	
Rural	30 (46.2)
Urban	35 (53.8)
**RODENTICIDE TYPE**	
Brodifacoum	29 (44.6)
Bromadiolone	36 (55.4)
Reason
Accidental	40 (61.5)
Suicide	8 (12.3)
Unknown^a^	17 (26.2)
**LABORATORY EXAMINATIONS**	
PT (seconds)	33.0 (36.1)
APTT (seconds)	83.3 (66.9)
**BLEEDING**	
Yes	36 (55.4)
No	29 (44.6)

*Data are number (%) or median (standard deviation)*.

a *Reasons for poisoning were unknown because exposure history was unavailable*.

### Demographics and clinical characteristics

Selected demographics and clinical characteristics of the high, medium, and low dose groups are shown in Table 3. There was no statistically significant difference in sex and environment (not shown in the table) among the three groups. Overall, patients in the high-dose had a wide age distribution, poisoned for various reasons, and arrived at the hospital later than 24 h (26/29, 89.7%), been misdiagnosed initially (25/29, 86.2%), not treated by gastric lavage (27/29, 93.1%), and developed severe hemorrhage. While most children in the low-dose were younger than 6 years (26/28, 92.9%), all have experienced accidental exposure, arrived at the hospital and received gastric lavage within 24 h, obtained a definite diagnosis, and be asymptomatic.

**Table 3:** Demographics and clinical characteristics of high, medium, and low dose group.

**Variable**	**High^a^*N* = 29**	**Medium *N* = 8**	**Low *N* = 28**	***p-*value**
**SEX**				
Male	13 (44.8)	5 (62.5)	17 (60.7)	0.449
Female	16 (55.2)	3 (37.5)	11(39.3)
**AGE (YEARS)**				
<6	15 (51.7)	3 (37.5)	26 (92.9)	^d^^e^<0.05
6 ≤ 10	6 (20.7)	1 (12.5)	1 (3.6)	
≥10	8 (27.6)	4 (50.0)	1 (3.6)	
**REASON**				
Accidental	7 (24.1)	5 (62.5)	28 (100)	<0.05
Suicide	5 (17.2)	3 (37.5)	0 (0)	
Unknown^b^	17 (58.6)	0 (0)	0 (0)	
**TIME FROM INGESTION TO HOSPITALIZATION (HOURS; DAYS)**				
<4 h	2 (6.9)	4 (50.0)	24 (85.7)	<0.05
4–24 h	1 (3.4)	2 (25.0)	4 (14.3)	
24 h−7 d	2 (6.9)	1 (12.5)	0 (0)	
≥7 d	24 (82.8)	1 (12.5)	0 (0)	
**TIME FROM INGESTION TO GASTRIC LAVAGE (HOURS)**				
<1	0 (0)	0 (0)	9 (32.1)	<0.05
1–4	2 (6.9)	4 (50.0)	14 (50.0)	
4–24	0 (0)	1 (12.5)	5 (17.9)	
No	27 (93.1)	3 (37.5)	0 (0)	
**INITIAL DIAGNOSIS**				
Definite	4 (13.8)	6 (75.0)	28 [100]	<0.05
Misdiagnosis	25 (86.2)	2 (25.0)	0 (0)	
**BLEEDING SITE**				
	3.14 (1.43)	1.63 (1.41)	0.04 (0.19)	<0.05
**ANEMIA**				
No	5 (17.2)	6 (75.0)	27 (96.4)	<0.05
Mild	5 (17.2)	0 (0)	1 (3.6)	
Moderate	15 (51.7)	2 (25.0)	0 (0)	
Severe	4 (13.8)	0 (0)	0 (0)	
**LABORATORY EXAMINATIONS**				
Hb (g/L)	80.8 (23.0)	114.5 (23.0)	123.1 (10.1)	^c^^d^<0.05
PT (seconds)	70.2 (42.2)	20.0 (19.9)	11.5 (1.0)	^c^^d^<0.05
APTT (seconds)	141.4 (55.7)	62.4 (46.2)	29.0 (5.1)	<0.05
INR	5.4 (2.1)	2.6 (1.8)	1.0 (0.1)	<0.05

*Data are number (%) or median (standard deviation)*.

a *High dose group: >24 points; medium: 15–24 points; low: <15 points*.

b *Reasons for poisoning were unknown because exposure history was unavailable*.

c *p < 0.05, the high-dose group vs. the medium-dose group*.

d *p < 0.05, the high-dose group vs. the low-dose group*.

e *p < 0.05, the medium-dose group vs. the low-dose group. PT, prothrombin time; APPT, activated partial thromboplastin time; INR, international normalized ratio*.

In the low-dose group, 24 of 28 patients usually consumed a few granules or had a “taste” of the rodenticides and did not develop coagulopathy. Although ingested large amounts (0.5–6 mL), the other four patients were also classified in the low-dose group and only one microscopic hematuria was observed. This might be because all of them were administered gastric lavage within an hour or plasmapheresis in the early phase, or both.

### Reasons for poisoning

As shown in Table 4, accidental exposure patients were younger than 6 years (34/40, 85.0%), and have taken baits (23/40, 57.5%). In 8 intentional suicide patients, 7 cases were older than 10 years, other one was at 8 years and 9 months old, and all of them have ingested the concentrates.

**Table 4:** Comparison of different reasons in children with 4-hydroxycoumarin intoxication.

**Variable**	**Accidental *N* = 40**	**Suicide *N* = 8**	**Unknown^a^*N* = 17**	***p-*value**
**AGE (YEARS)**				
<6	34 (85.0)	0 (0)	10 (58.8)	^b^^d^<0.05
6 ≤ 10	3 (7.5)	1 (12.5)	4 (23.5)	
≥10	3 (7.5)	7 (87.5)	3 (17.6)	
**AVAILABLE FORMS**				
Bait	23 (57.5)	0 (0)	0 (0)	<0.05
Concentrate	17 (42.5)	8 (100)	0 (0)	
Unknown	0 (0)	0 (0)	17 (100)	
**TIME FROM INGESTION TO HOSPITALIZATION (HOURS; DAYS)**				
<4 h	28 (70.0)	2 (25.0)	0 (0)	<0.05
4–24 h	6 (15.0)	0 (0)	0 (0)	
24 h−7 d	3 (7.5)	1 (12.5)	0 (0)	
≥7 d	3 (7.5)	5(62.5)	17 (100)	
**TIME FROM INGESTION TO GASTRIC LAVAGE (HOURS)**				
<1	8 (20.0)	0 (0)	0 (0)	^b^^c^<0.05
1–4	18 (45.0)	2 (25.0)	0 (0)	
4–24	7 (17.5)	0 (0)	0 (0)	
No	7 (17.5)	6 (75.0)	17 (100)	
**INITIAL DIAGNOSIS**				
Definite	35 (87.5)	2 (25.0)	1 (5.9)	^b^^c^<0.05
Misdiagnosis	5 (12.5)	6 (75.0)	16 (94.1)	

*Data are number (%) or median (standard deviation)*.

a *Reasons for poisoning were unknown because exposure history was unavailable*.

b *p < 0.05, the accidental exposure group vs. the suicide group*.

c *p < 0.05, the accidental exposure group vs. the exposure history unknown group*.

d *p < 0.05, the suicide group vs. the exposure history unknown group*.

Six of eight intentional suicide patients concealed what they ingested and 17 patients had no definite history of ingestion. These patients were more likely to arrive at the hospital after 24 h (suicide 6/8 and unknown 16/17), experience higher incidence of misdiagnosis (suicide 6/8 and unknown 16/17), thus not receive gastric lavage treatment (suicide 7/8 and unknown 17/17), and eventually be divided to high (suicide 5/8 and unknown 17/17), or medium (suicide 3/8) dose group than accidental exposure were. Moreover, since the parents of 5 accidental exposure patients did not realize the risk of consuming the agent, neither did provide exposure history to physicians on admission, their kids were also misdiagnosed initially, and were divided to high (4/5) or medium (1/5) dose finally. Of 27 misdiagnosed patients, 11 were misdiagnosed once, another 11 misdiagnosed twice, 2 misdiagnosed thrice, and remaining 3 misdiagnosed more than thrice. No exposure was identified in a one-year-old boy, leading to misdiagnosis with hemophilia. And it took 1 year to make a definite diagnosis using LC-MS/MS method.

### Predictability of TDCH

Firstly, the AUROC, Youden index, sensitivity, and specificity were calculated to determine the predictability for coagulopathy of TDCH (Table 5). Of 38 patients arrived at hospital within 48 h, patients a score_48h_ ≥ 15 had higher incidence of coagulopathy (6/8, 75.0%) than patients with a score_48h_ < 15 (3/30, 10.0%). The AUROC, Youden index, sensitivity and specificity were 0.912, 0.727, 0.727, 1.000, respectively, which showed middle sensitivity and high specificity in predicting for coagulopathy of TDCH. 27 patients who arrived at hospital after 48 h all presented with coagulopathy when admitted. So we did not use TDCH to predict coagulopathy in these patients.

**Table 5:** Predictability of TDCH.

	**AUROC**	**SE**	***p* value**	**Asymptotic 95% confidence**		**Cutoff point**	**Youden index**	**Sensitivity**	**Specificity**
				**Lower bound**	**Upper bound**				
Coagulopathy^a^	0.912	0.058	0.000	0.798	1.000	15	0.727	0.727	1.000
Prolonged administered with vitamin ${\text{K}}_{1}^{\text{b}}$	0.995	0.005	0.000	0.985	1.000	15	0.933	1.000	0.933

a *TDCH score_48h_ (n = 38) were predictive for coagulopathy*.

*^b^TDCH scores (n = 65) were predictive for prolonged administered with Vitamin K_1_ (≥ 1 month). AUROC, area under the receiver operating characteristic; SE, standard error*.

Secondly, the predictability for prolonged administration with vitamin K_1_ (≥ 1 month) of TDCH was analyzed by AUROC. Of all patients, 37 in the high and medium dose group with a score ≥ 15 has higher incidence (35/37, 94.6%) of prolonged administration with vitamin K_1_ (≥ 1 month) than other 28 patients with a score < 15 (0/28, 0%). The AUROC, Youden index, sensitivity and specificity were 0.995, 0.933, 1.000, 0.933, respectively. The results above showed high predictability for prolonged administration with vitamin K_1_.

## Discussion

We introduced a novel evaluation system for the determination of toxic dose in children with 4-hydroxycoumarin rodenticide intoxication. In this study, deliberate suicide patients (8/8, 100%) presented with obvious hemorrhage and were divided to high or medium dose group, while (28/40, 70%) accidental exposure patients were often asymptomatic and were divided to low dose. Our observations were consistent with an analysis of the American Association of Poison Control Centers' National Poison Data System (NPDS) showing that intentional exposures had a greater frequency of serious outcomes than unintentional exposures did (11). In addition, we found that 17 patients whose exposure information was unavailable all presented with severe hemorrhage and were divided to high dose. Interestingly, 18 patients younger than 6 years were divided into the high or medium group in our study. They presented with coagulopathy, inconsistent with a previous study showing that preschool-aged children with unintentional acute exposure to superwarfarin rodenticides would not likely develop any significant coagulation abnormalities (12). Of the 18 patients, ten denied ingestion of rodenticides and, therefore, they were not administered with gastric lavage. Five with accidental exposures ingested large amounts of concentrations (ranging from 0.5 to 2.5 mL), and three of the five subjects were administered with gastric lavage within 4 h. The remaining three with accidental exposures ingested unclear amounts and only one of them received gastric lavage. Ingestion of large amounts and delayed treatment may be the main causes of coagulopathy in these patients.

Misdiagnosis is the most common clinical challenge of 4-hydroxycoumarin rodenticide poisoning. It mainly occurred both in intentional suicide patients (6/8, 75.0%) and those who had no definite history of ingestion (16/17, 94.1%). The key element of diagnosis, the history of ingestion, is often unavailable in these patients. As a result, misdiagnosis and delayed treatment are common, often leading to severe consequences. 4-Hydroxycoumarin rodenticide poisoning is usually misdiagnosed as hemophilia when it occurs in little children without a definite history of ingestion. Because the onset is insidious, the hemorrhage is prolonged and recurrent, and factors IX and X is decreased. Anticoagulant rodenticides poisoning should be considered in cases of unexplained multi-site bleeding and vitamin K-dependent coagulation disorder. The key to avoiding misdiagnosis is to obtain a clear history of rodenticides ingestion or have access to a toxicology screen, such as LC-MS/MS.

The TDCH could evaluate toxic doses, predict coagulopathy in the early and guide the administration of vitamin K_1_. Limited studies have shown that a child weighing 10 kg would need to acutely ingest a minimum of 0.15 mg/kg of brodifacoum for coagulopathy to develop (13). However, four of our patients ingested large amounts of rodenticides, but they did not present with obvious coagulopathy because of the timely gastric lavage and plasmapheresis. Thus, the amount ingested could not be predictive of coagulopathy. Using TDCH, we found patients with a score_48h_ ≥ 15 were in the high bleeding risk category. Early identification of these patients is crucial for not only promoting precise early treatment of high-risk patients, but also avoiding unnecessary examinations and treatments in those with low risks. For high-risk patients, vitamin K_1_ should be administered preventively as soon as possible. Up to now, there was no clear consensus on the appropriate use of vitamin K_1_ (14). According to a literature review (7), the duration of vitamin K_1_ ranged from 28 days to 730 days. And in our observation, patients with obvious coagulopathy required vitamin K_1_ at least for one month because of ongoing bleeding or coagulopathy. So we chose longer than a month as the length of vitamin K_1_ treatment. We found that patients in the high and medium dose with TDCH scores ≥ 15 required treatment with vitamin K_1_ for more than a month. With an AUROC of 0.995 and sensitivity of 0.933 and specificity of 1.000, the TDCH has high predictability.

However, this study still has some limitations. First, most diagnoses were based on the history of exposure to the rodenticides without qualitative identification of 4-hydroxycoumarin. Qualitative identification of 4-hydroxycoumarin in the blood was performed only in some patients, and none of the patients were subjected to quantitative analysis of the toxin. But this measurement is not currently widely performed even in the developed countries. Quantitative analyses, which can objectively reflect the toxic *in vivo* dose, are gold standard criteria for diagnosis. The TDCH could be further developed by using quantitative analyses. Second, the number of patients was insufficient in our study, and the TDCH score should be further verified and improved in larger, prospective studies.

## Conclusion

The TDCH system is not only suitable for evaluating toxic doses on admission and predicting the risk of coagulopathy in the early stage, but also helps to appropriate treatment. Using TDCH, we found patients with a score_48h_ ≥ 15 were in the high bleeding risk category. And patients with a scores ≥ 15 required treatment with vitamin K_1_ for more than a month. It is a simple and non-invasive method, and can be widely used in all areas, especially in developing countries. We hypothesize that modified TDCH can also be applied to other intoxication in children.

## Author contributions

ZW designed and conducted the studies, edited the manuscript, PI of the funding; LY collected and analyzed the data, and drafted the manuscript; HZ and HG collected the data and edited the manuscript; YG and LC analyzed the data and edited the manuscript. All authors approved the final manuscript.

### Conflict of interest statement

The authors declare that the research was conducted in the absence of any commercial or financial relationships that could be construed as a potential conflict of interest. The reviewer CO and handling Editor declared their shared affiliation.

## References

[1] Hadler MR Shadbolt RS. Novel 4-hydroxycoumarin anticoagulants active against resistant rats. Nature (1975) 253:275–7. 10.1038/253275a0 1113846

[2] Lipton RA Klass EM. Human ingestion of a 'superwarfarin' rodenticide resulting in a prolonged anticoagulant effect. JAMA (1984) 252:3004–5. 10.1001/jama.1984.03350210052030 6502864

[3] Sarin S Mukhtar H Mirza MA. Prolonged coagulopathy related to superwarfarin overdose. Ann Intern Med. (2005) 142:156. 10.7326/0003-4819-142-2-200501180-00024 15657170

[4] Sharma P Bentley P. Of rats and men: superwarfarin toxicity. Lancet (2005) 365:552–3. 10.1016/S0140-6736(05)70773-X 15708084

[5] Hellemans J Vorlat M Verstraete M. Survival time of prothrombin and factors VII, IX and X after completely synthesis blocking doses of coumarin derivatives. Br J Haematol. (1963) 9:506–12. 10.1111/j.1365-2141.1963.tb05475.x 14076133

[6] Ingels M Lai C Tai W Manning BH. A prospective study of acute, unintentional, pediatric superwarfarin ingestions managed without decontamination. Ann Emerg Med. (2002) 40:73–8. 10.1067/mem.2002.125449 12085076

[7] Caravati EM Erdman AR Scharman EJ Woolf AD Chyka PA Cobaugh DJ. Long-acting anticoagulant rodenticide poisoning: an evidence-based consensus guideline for out-of-hospital management. Clin Toxicol. (2007) 45:1–22. 10.1080/15563650600795487 17357377

[8] Patocka J Petroianu G Kuca K Toxic potential of superwarfarin: Brodifacoum. Mil. Med. Sci. Lett. (2013) 82:32–7.

[9] Schaff JE Montgomery MA. An HPLC-HR-MS-MS method for identification of anticoagulant rodenticides in blood. J Anal Toxicol. (2013) 37:321–5. 10.1093/jat/bkt036 23667199

[10] Bolton-Maggs P Moon I. Assessment of UK practice for management of acute childhood idiopathic thrombocytopenic purpura against published guidelines. Lancet (1997) 350:620–3. 10.1016/S0140-6736(97)04143-3 9288044

[11] Mowry JB Spyker DA Brooks DE Zimmerman A Schauben JL. 2015 Annual Report of the American Association of Poison Control Centers' National Poison Data System (NPDS): 33rd Annual Report. Clin Toxicol. (2016) 54:924–1109. 10.1080/15563650.2016.1245421 28004588

[12] Mullins ME Brands CL Daya MR. Unintentional pediatric superwarfarin exposures: do we really need a prothrombin time? Pediatrics (2000) 105:402–4. 10.1542/peds.105.2.402 10654963

[13] Smolinske SC Scherger DL Kearns PS Wruk KM Kulig KW Rumack BH. Superwarfarin poisoning in children: a prospective study. Pediatrics (1989) 84:490–4. 2771552

[14] King N Tran MH. Long-acting anticoagulant rodenticide (superwarfarin) poisoning: a review of its historical development, epidemiology, and clinical management. Trans. Med. Rev. (2015) 29:250–8. 10.1016/j.tmrv.2015.06.002 26239439

